# Negotiating scientific knowledge in the development of an eHealth MOOC

**DOI:** 10.1007/s10639-022-11107-3

**Published:** 2022-05-24

**Authors:** Heidi Gilstad, Martha Skogen, Pieter Toussaint, Cathrin B. Larsen, Arild Faxvaag

**Affiliations:** 1 SEKOM, Department of Language and Literature, NTNU-Norwegian University of Science and Technology, Trondheim, Norway; 2 Department Rheumatology, St. Olavs University Hospital, Trondheim, Norway; 3 Department of Neuromedicine and Movement Sciences, NTNU, Trondheim, Norway; 4 Department of Informatics and Computer Science, NTNU, Trondheim, Norway; 5 Technology Management, SINTEF, Trondheim, Norway

**Keywords:** Scientific communicative competence, eHealth, MOOC, Educational tools, Interdisciplinary, Expertise, Dialogue, Discourse, Visual communication tools, Conceptual framework, Collaborative reasoning

## Abstract

Interdisciplinary team communication in eHealth development is challenging because all disciplines have unique, intrinsic discursive practices, theories and artefacts. Due to these factors, members of interdisciplinary teams can experience problems in communication and collaboration. Through a centered focus, members can benefit individually, inspire one another, and ultimately reach a timely delivery of their common pedagogical goal(s). Using the lens of dialogism, this paper aims to identify the conceptual considerations that arose during the development of a Massive Open Online Course (MOOC) for higher education in eHealth. Methods included auto-ethnography and interdisciplinary dialogue supported by literacy artefacts, including visual material. Results yielded a visual tool for meta-assessment of team communication, and an organizing principle for topics in the MOOC. A major implication is that especially for eHealth, scientific communicative competence of experts—while establishing a common understanding—can lead to a unique and meaningful delivery of high pedagogical quality.

## Introduction

Interdisciplinary work can result in complex problem solving and innovation, yet also be discursively complicated for participants, especially when they cover a wide range of expertise. In scientific teamwork, each participant represents a discipline where scientific discourse evolves through communicative cooperation between experts in the field. Ideas are shared with the aim of developing knowledge and positioning the discipline in relation to other disciplines. Language and discourse are essential in creating and maintaining professions and disciplines (Gunnarsson, 1997). When experts from different scientific disciplines work together, they must adjust communicatively to each other to be able to share and understand insights relevant to the topics of the project and to the relevant context(s) and discourses. The degree to which they do so affects the momentum of the group, as well as achieving a successful outcome.

Teamwork regarding eHealth development is particularly complicated, as eHealth solutions are complex and meant to serve different purposes for different actors in different contexts (Pagliari, 2007; Van Velsen et al., 2013). EHealth solutions include digital information, communication, reporting, documentation, and learning solutions regarding health for citizens, patients, health personnel, health management, and administration. Depending on the purpose and functionality of the eHealth solution, insights gained through productive interdisciplinary teamwork may inform the solution’s health content, communication, design and technology. Especially for eHealth pedagogical tools, the inclusion of interdisciplinary input ensures that broad demands on human and technological aspects are adequately addressed.

In «The Reflective Practitioner», Schön (1983) dicussed the working practices and mindsets of engineers, architects, managers, psychotherapists, and city planners, etc. He observed that the professionals met challenges both by *reflecting- in- action* and *reflecting-on action*, and that they improved the work and learned by reflecting on their practices. In this paper, five practicing researchers, representing applied linguistics, medicine, informatics, design and nursing, go from being discipline-specific reflecting researchers to an becoming an interdisciplinary reflecting team during the development of a massive open online course (MOOC) for healthcare professionals on a master student level.

This study aimed to investigate interdisciplinary communication and conceptual considerations during the development of a MOOC in the Smart Digital Health Communication project (2017). The study posed two research questions:

- *RQ1: Which conceptual considerations arose during the development of the MOOC?*
- *RQ2: What characterized the communication between the experts in the interdisciplinary team?*

This paper increases knowledge about interdisciplinary eHealth MOOC development as it was experienced by a team of experts. The contributions include awareness of metaperspectives of interdisciplinary collaboration and strategies for how to accomplish common goals.

## Theoretical perspectives on communication and interdisciplinary collaboration

This paper adopts a dialogic, interactional approach to language and communication, while focusing on the professional discourse in an interdisciplinary research and development project and MOOC for education in diverse aspects of eHealth.

### Dialogism and interdisciplinary professional discourse

*Dialogism* is a theoretical approach that presents a perspective on how human interactions can be understood (Linell, 2009). Humans are social creatures that depend on other human beings, materially and for existential growth and development (Berger & Luckman, 1966). Dialog is essential for reasoning, learning, knowledge development, and innovation. Thinking, meaning-making, language use, utterances, signs, and text are *dialogical*, with an imaginary recipient and an expectation of response, *polyvocal*, referring to different voices, and *referential*, indicating carriers of information about the world (Bostad et al, 2004). This perspective on human interaction has consequences for the study of language, communication, and discourse. Importantly, contextual aspects influence human interactions. Moreover, the *communicative competence* of the participants, ability to use language adequately in a social context, is important for the interpretation of the utterances, be they spoken, written or multimodal (Hymes, 1972). In this paper we understand *discourse* as meaning-making in context, more specifically as a unity of ways of saying (informing), doing (action), and being (identity) through language (Gee, 2014).

Professional discourse and practice are increasingly specialized, and interdisciplinary interaction is required to solve complex tasks and challenges (Sarangi, 2005). However, interdisciplinary communication may be complicated, as different disciplines and professions have different focus areas. Goodwin (1994) suggested that experts develop a professional vision, that is, a way of seeing the world through the lenses of the knowledge and practices of their particular discipline. Discursive practices in the respective discipline evolve over time, defining the objects of knowledge, such as theories and methods, as well as practical conduct and procedures of the discourse community (Hyland, 2011). Expertise may be explicitly defined in literacy artefacts such as procedures and job descriptions (Pahl & Rowsell, 2010), while the expert professional conduct has dimensions that are tacit, i.e., experience-based and internalized, but difficult to explain (Polanyi, 2002).

**Fig. 1:**
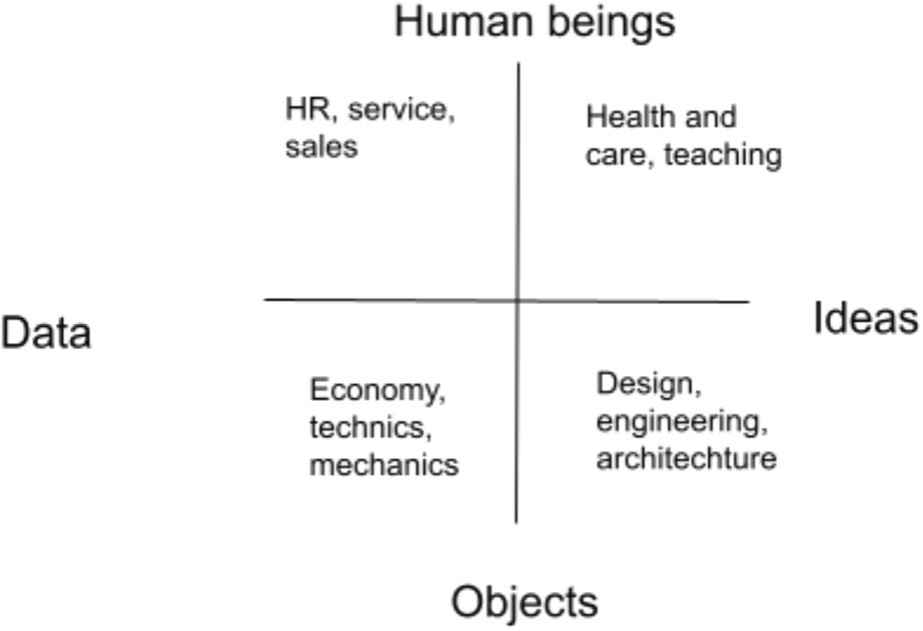
Focus areas in professional practice, including examples ((Anna-Malin Karlsson, 2006). Our translation. Permission granted by author)

Karlsson (2006) suggested that while some professions focus on humans, others focus on data, objects, or ideas (Fig. 1).

The focus area and professional vision of the practitioner have consequences for professional conduct. Moreover, the organization of the team or community impacts the communication. Teams can be defined as *groups of two or more individuals working interdependently toward a shared goal that requires coordination of effort and resources to achieve mutually desired outcomes* (Salas et al., 1992). The definition is helpful, but lacks an emphasis on the contextual resources and the communicative expertise required to be able to collaborate.

In this paper, we apply the term discourse community as a point of departure for discussing the work organization and communication of the group in context*.* Primarily concerned with the written academic discourse, linguist John Swales defined discourse communities as “groups that have goals or purposes, and use communication to achieve these goals” (Swales, 2011). Another, but still related, group phenomena is the *community of practice*. The *practice* aspect is essential. Communities of practice in which expertise is developed have different characteristics regarding organization and tasks and roles of the participants (Lave & Wenger, 1991). While some communities of practice may be temporary and task-oriented, such as project-based teams, others may be organized in stable communities that evolve over time, such as workplaces or scientific disciplines.

A team consists of members who have the competencies necessary to fulfill objectives and tasks. Team members have different roles, the most common being management (responsible for project progress), production of tasks, decision-making (alone or with others), and interested persons who must be consulted, may be consulted, or must be informed (Anderson, 2016). The organizing principle of a team varies depending on its purpose and goals. Four types of team organizations may be distinguished: unidisciplinary, interdisciplinary, multidisciplinary, and transdisciplinary (Lotrecchiano, 2010, 2020). These shift from singular persons bringing in histories, traditions, and expectations in relatively closed and not interdependent ways, to holistic fusion of histories and expectations in reciprocal interdependencies between the participants. Klein (2018) elaborated on the organizing principles of teams and suggested that interdisciplinarity (ID) “integrates information, data, methods, tools, concepts, or theories from two or more disciplines or bodies of knowledge in order to address a complex question, problem, topic, or theme. Solo interdisciplinarians work independently, but communication across boundaries is essential to "collaboration”.

More fused knowledge exchange is transdisciplinarity (TD). According to Klein (2018), transdisciplinarity transcends disciplinary worldviews by generating overarching synthetic frameworks and by engaging stakeholders in co-production of knowledge. Transdisciplinarity connotes teamwork aimed at generating new conceptual and methodological frameworks (Klein, 2018).

Communication in inter- and transdisciplinary teams is complex and requires collaborative reasoning, among other factors. Our claim is that each participant must have *scientific communicative competence*. Referring to the health context, Sarangi (2005) observed that healthcare professionals may be proficient in scientific knowledge but not the communicative dimension. The same applies to participants in interdisciplinary scientific communication. Communicative competence required in interdisciplinary teamwork includes the ability to share a temporary, shared social reality (Rommetveit, 1974). This includes the ability to listen carefully, try to capture other scientific insights, and to express significant points from their own scientific discourse in a jargon that displays the topics adequately but also understandably for the addressee (Gilstad, 2021). As Dysthe et al. (2000, 155–156) suggest, interdisciplinary teams should consider scientific disagreement as a strength and as an idea generator. They warn about discussions where the point is to “win” the discussion.

The organization of the team depends on the degree of commitment of the participants. In knowledge-contexts, such as universities, people are driven by scientific interests. However, conflicts of interest, clashes of discourses, and various degrees of scientific respect remain across scientific disciplines and methodologies. Consequently, interdisciplinary research requires tolerance (Locker, 1994). Likewise, development of interdisciplinary educational tools, as written or multimodal texts, requires the ability to pragmatically assess own practices and to try out alternative perspectives (Dysthe et al., 2000).

## Methods

This study adopted an ethnographic approach for the ongoing and retrospective assessment of team communication, inspired by the notions of *thick description* (Geertz, 1973) and *thick *participation (Sarangi, 2005). A thick description is an ample reporting of the phenomena observed, often including an introspective reflection of the researcher. Thick participation describes the researchers’ role when they immerse themselves into the context they study. In this project, the team members were both researchers and informants who contributed to the meta-assessment with individual perspectives on team communication, based on field notes and team discussions.

### The scientific profile of the team

The team consisted of five professionals with different scientific profiles: an associate professor in applied linguistics, a researcher and associate professor with a PhD in design and visual communication, a professor in informatics, a nurse and PhD student in health communication, and a professor in health informatics and rheumatology. Although the disciplines represented were primarily academic, the disciplines were distinctly different in terms of the epistemological points of departure (positivist vs. socio-constructivist), scientific and methodological orientation (quantitative vs. qualitative), and knowledge-approach (procedural, know-how, practice-oriented vs. propositional, know-what, theory-oriented). *Applied linguistics* is a problem-driven discipline (Evensen, 2013), which adopts dialogic,interactionist approaches  to study language and communication practices (speech, text, multimodality, and ethics in interaction) between human beings (and technologies) in real situations and contexts. *Visual communication design* is an empirically-oriented discipline, with sociocultural and user-centered approaches to study the design of solutions, objects, tools, and processes. This study focused primarily on human and digital interactions which included the multimodality of text, images, and visualizations. *Informatics* is a technology- and process-oriented discipline with positivist and socio-constructivist roots. This study primarily investigated technological architecture, processes, and use, including programming, codes, and terminologies. *Medicine* is a practice-oriented theoretical discipline with a positivist approach to science and an approach to the human body as an object that can be observed, understood, and changed. A socio-constructivist perspective on holistic human beings as biology and mind has become increasingly accepted. *Nursing* is practice-oriented with a holistic approach to human beings. The epistemological point of departure is positivist but increasingly influenced by socio-constructivist approaches. Ethical considerations and communication are part of practical and theoretical training Table 1.

**Table 1:** Description of the experts in the team

Expert	Gender	Age	Specialization	Nationality	Role in the project	Years of teaching experience	Experience with MOOC development	Experience with MOOC use
HG	F	53	Applied Linguistics, Health Communication and Literacy	Norwegian	Project Manager	10	no	some
AF	M	62	Health informatics, Consultant in Rheumatology	Norwegian	Participant	28	no	some
PT	M	56	Information Systems, Health Informatics	Dutch	Participant	15	no	some
MRS	F	54	Design	American/Norwegian	Researcher	5	some	some
CBL	F	32	Nurse, Global Health, Health Communication	Norwegian	PhD-student	0	no	some

The diversity between epistemological points of departure, theoretical perspectives, methodological aspects, and knowledge areas forced the group to be explicit about their insights and attentive to other perspectives. Successful interactions were rooted in a shared awareness of curiosity and mutual respect. All members understood that one common goal was to develop an interdisciplinary eHealth MOOC for university level students. Collaborative work led directly to generation of high-quality, customized pedagogical material. The overall success of the project depended on the team’s communicative interactions and the timely delivery of the MOOC.

### Distribution of work

**Fig. 2:**
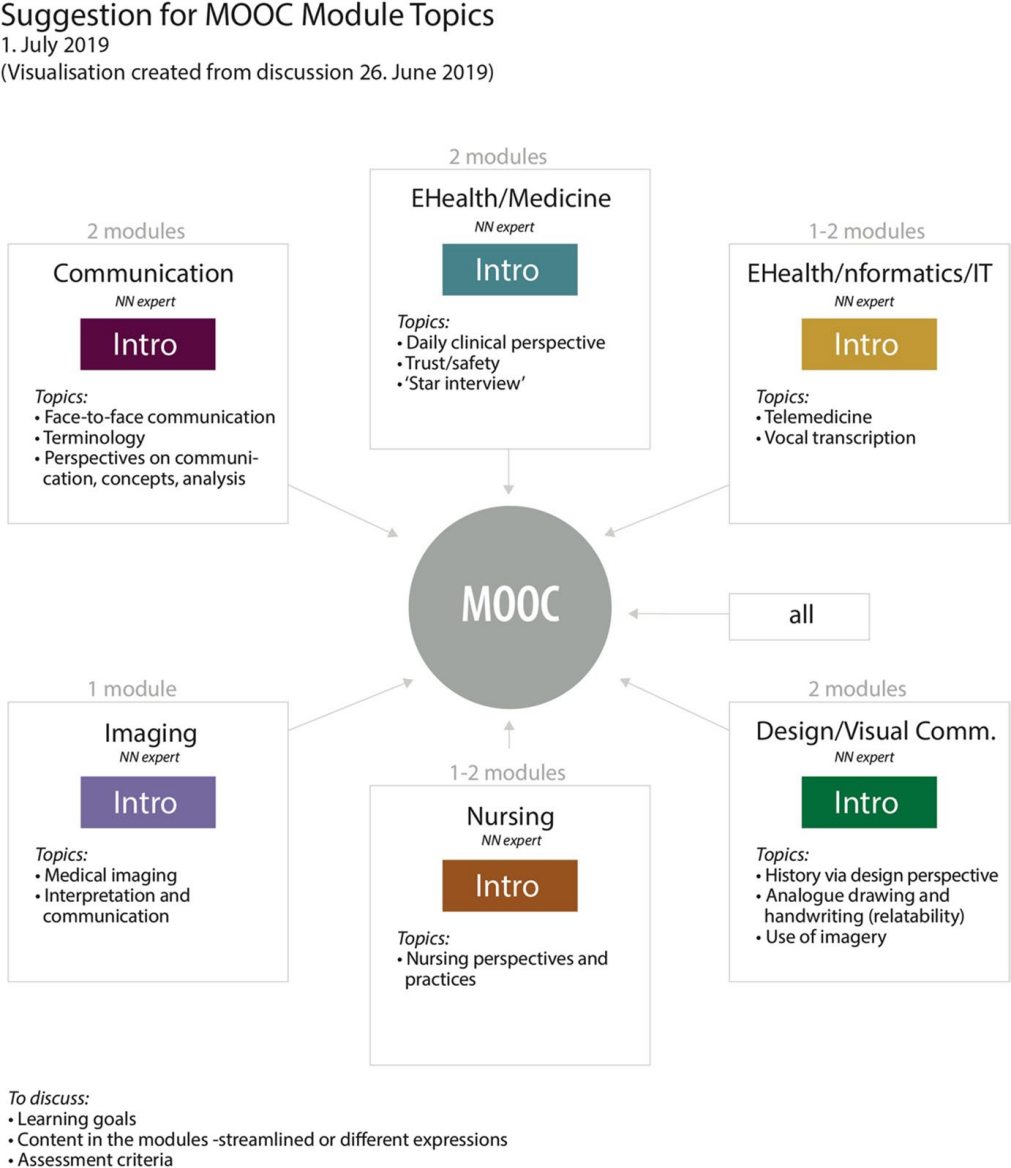
First version of the visualization process. This map shows each topic with its associated expert and initial topics of interest listed underneath. Note the mixed languages, as three cultures across two continents were included. Names have been covered for privacy. (Martha Skogen 2019. Permission granted by author)

The process of developing the conceptual framework for the MOOC was driven by the applied linguist and designer in designated seminars. In intertextual and interdiscursive (Koskela, 2013) processes, the team referred to previous discussions and condensed the most prevalent topics and concepts. The first outline of visualization of the topics, the “mandala” (Fig. 2), was discussed by the team, resulting in a revision of the bridging concepts. After several iterations, the mandala was finalized and became the organizing principle for the MOOC. Each team member was responsible for their topic and developed educational content in accordance with the agreed learning material, structure, and principles of learning outcomes for the students. Parallel to this study, the first author created the theoretical framework and structural organization of the text, inviting others to contribute substantially to all parts of the text, particularly what fell within their own areas of expertise. The visual design of the mandala attempted to unify and organize the team’s insights.

### Interdisciplinary expert dialogue

Meetings of the interdisciplinary group took place twice a month over a period of four years. Regular meetings with a semi-structured agenda allowed for reflective discussions and instrumental task work. Such an approach allows for discussions of overall topics as well as clarifications and expansion of subtopics, notions, and concepts when they occur. While professional work is often task-oriented and time-restricted, interdisciplinary dialog in this group allowed for formal and informal exchange of ideas within the time frame of the meeting. Regular meetings every two weeks allowed for follow-up on previous discussions and references to previous concepts, consequently leading to knowledge-sharing and learning within the group. This culture of references to previous talk and texts is an example of an intertextual and interdiscursive approach (Koskela, 2013), where new thoughts are explicitly linked to previous knowledge exchange in the group. The group members brought in experiences and activities from other forums.

The work process of the group evolved over time and included the phases shown in Table 2.

**Table 2:**
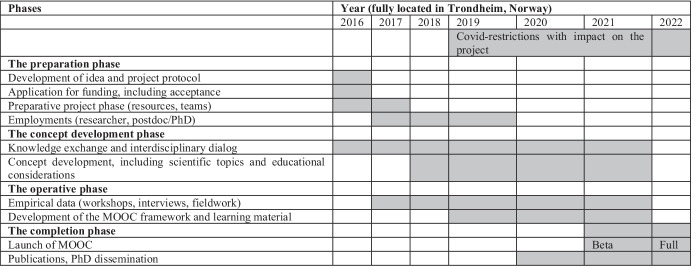
The main phases of the project

During the concept development phase and the operative phase, the collaboration of the team evolved in a semi-structured manner, with ongoing collaborative interdisciplinary reasoning, applying collaborative communicative competence. As the concepts and topics were agreed upon, individualist and operative approaches were adopted, with individual experts responsible for developing specific learning materials for the MOOC. The MOOC building was a central focus point. We were able to complete its delivery due to our understanding that all disciplines should be represented in equal measure.

### Empirical data

The interdisciplinary dialogue and knowledge exchange was nurtured by the parallel collection of empirical data throughout the process. With mixed methods, data about user participation and involvement, health language and health literacy was collected.

## Analysis

The ethnographic approach of thick description, (Geertz, 1973), was combined with auto-ethnography (Anderson, 2006), which has five key features: complete member researcher status, analytic reflexivity, narrative visibility of the researcher’s self, dialogue with informants beyond the self, and commitment to theoretical analysis. The autoethnographer, as a member of a group and as a researcher, develops understanding from engaging in dialogue. Autoethnography includes analytic reflexivity on the reciprocal connection between the person, the other participants, and the environment in which the communication takes place. The researcher reflects introspectively, while also in interaction with the others (as this paper is an example of), and as such positions and presents herself/himself in relation to the other participants. Based on recurring analytic reflexivity, introspection and dialogue with others, the participants may come to a broader understanding of the communicative and social phenomena. (Anderson, 2006).

**Fig. 3:**
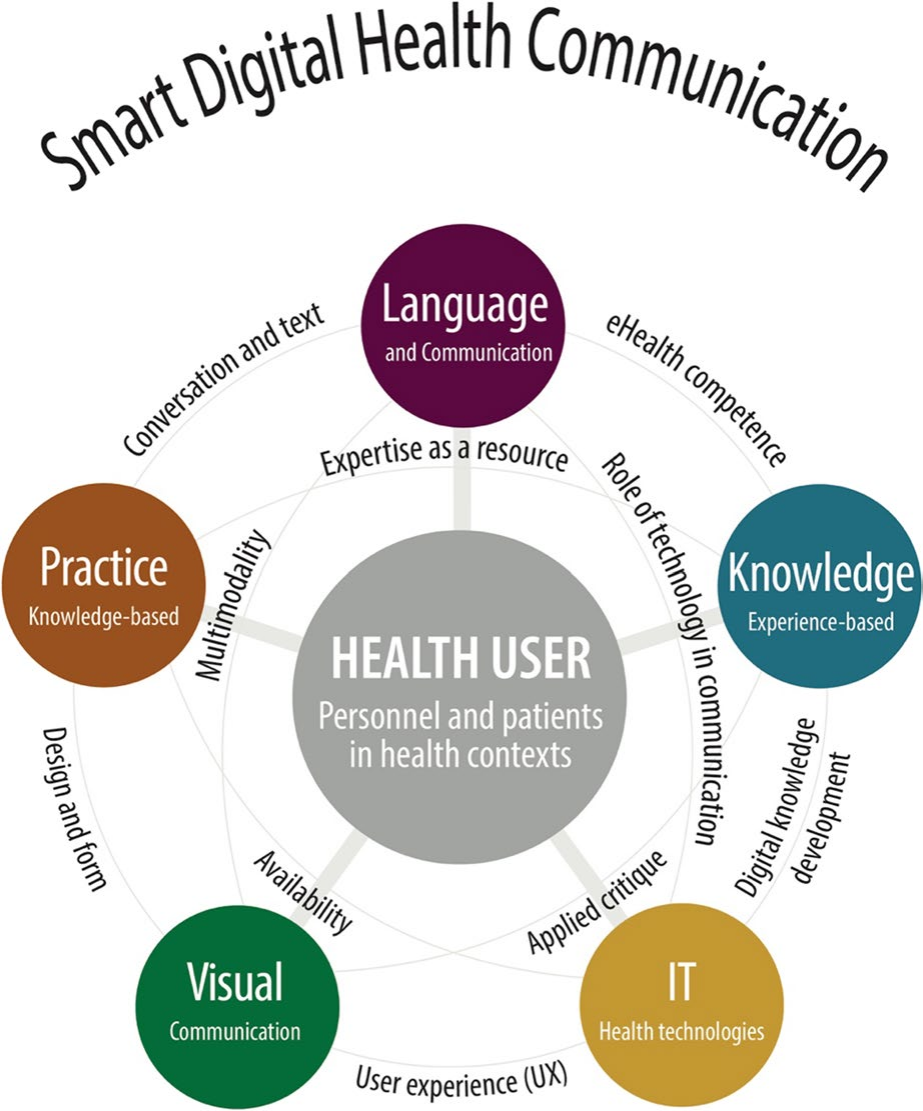
The “mandala”: a customized visual tool for representing and understanding interdisciplinary relationships (Martha Skogen, 2019. Permission granted by author)

The analytic reflexivity in the group work involved both structured and unstructured dialogue. The experts reflected individually on their contribution, and in the ongoing dialogue, communicated their scientific insights. The expert knowledge provided the outline of analytic categories (Fig. 2), and in a recurring deduction was organized in themes (Fig. 3). In the following sections, the respective scientific considerations are presented.

### Developing a conceptual framework for the MOOC

With dialogic perspective on language, learning, and communication serving as a points of departure, the interdisciplinary group categorized the knowledge and deduced analytic themes. Team discussions resulted in the topics' relevant inclusion in the MOOC. Based upon the team’s expertise and learning goals that were defined for a MOOC in digital health communication, the following topics were suggested as the main topics: *language and communication, experience-based knowledge, knowledge-based practice, IT health technologies, and visual communication.* These five topics were motivated by the expert knowledge and insights of the group, as well as by empirical work (see Table 3). In the following section, we elaborate on the five topics and related subtopics.

**Table 3:** Empirical data that nurtured the interdisciplinary dialogue

Participants	Methods	Informants	Number	Topic	Place (Norway)	Year
Project manager (PM) and postdoc (PD)	Workshop	User representatives	40	Patient experiences, preferences and obstacles with digitalization	Hell	2017
PM&PD	Workshop	User representatives	25	As above	Molde	2018
PM&PD	Workshop	Healthcare professionals	25		Trondheim	2018
PM	Semi-structured interview	Patients (lupus)	8	Patient narratives	Trondheim	2018
Researcher	Pilot interviews	Healthcare professionals	10	Visual communication tools during face-to-face consultations	Trondheim	2019 (follow-up in 2020 was cancelled due to Covid-19)
PhD-candidate	Fieldwork, video-recordings of face-to face consultations	Nurses and patients	15	Health literacy	Trondheim	2020–2021 (pilot phase and data collection)
All	Semi structured group talk with advisory board	User representative and external experts	8	Project design		2016–2021
All	Unstructured workshop with digital learning group at the university		4	MOOC design, learning goals		2018–2021

### Topic: Language and communication

Contexts and contextual resources are decisive in conveying the spoken and written utterances, and their potential interpretations (Linell, 2009). But what characterizes the different modes?

*Spoken language* is traditionally characterized as aural and transient, produced and processed in real time with limited opportunity to plan, usually permitting immediate interaction where the speaker and hearer share a spatiotemporal context (Cameron & Panović, 2014). W*ritten language* is visual and more permanent, can be extensively planned, edited, and processed in different ways, does not permit immediate interaction, and the writer and reader do not need to share spatiotemporal context (Cameron & Panović, 2014).

Focusing on communication in health contexts, Gilstad (2021) offers a definition of the term *health language*, and suggests that it is “linguistic communication in speech, writing or signs about health and illness in a professional health context”. Practitioners in the healthcare system, such as nurses, doctors, and physiotherapists, routinely talk with each other and the patients. Talk allows for dynamic initiative and response between the addresser and the addressee in the context, and the participants may repair misunderstandings synchronously (Schegloff et al., 1977).

Traditionally, health language took place in physical face-to-face situations. However, currently, health language increasingly takes place in digital contexts (Hem & Nylenna, 2021). Documentation, reporting, and registration are crucial communicative activities in healthcare systems. Moreover, written letters, messages, medical notes, patient records, prescriptions, and medical information are communicated to patients and citizens. Written communication is both, legally required and medically important (Nordrum, 2021).

In interaction, we apply verbal and non-verbal sign systems, adapted to addressees and socio-contextual prerequisites (Gumperz, 2009). Sign systems are multimodal. Multimodality can be a combination of talk and signals with eyes, or gestures, or a complex whole of verbal, non-verbal, visual, auditory, tactile, and digital signs.

Health communication is evolving in the eHealth era (Neuhauser & Kreps, 2003), and patients have access to diverse health information. eHealth communication and eHealth information exchange are distinctly different activities. The key differences are comparable with the difference between spoken and written language. We refer to *eHealth communication* as technology-mediated spoken or sign-based communicative activity, where the uttered messages are exchanged between two or more actors, and where there is a temporal opportunity to respond to the utterance in the specific context.

Contrastingly, we understand *eHealth information exchange* as the context-specific, technology-mediated exchange of written and multimodal literacy artefacts, such as videos and images, documents, forms, procedures, reposts, and plans with an asynchronous means for feedback.

In addition to other contextual resources, the modality has consequences for the interpretation of the communicated message (Hutchby, 2001).

To interpret and understand the communicated message and information exchanged in the digitalized healthcare system, the user needs a certain level of eHealth literacy (Norman & Skinner, 2006). The eHealth literacy level in a population varies and depends on general literacy (reading, writing, numeracy) and the ability to apply digital devices purposefully and act thereafter. Moreover, socio-contextual, and cultural aspects influence the eHealth literacy level of citizens (Griebel et al., 2018). Due to the variety of literacies, the providers of eHealth communication and information need to provide content that is commonly understandable. In order for citizens to understand and act on health-related information, it should be adapted meaningfully in the context and presented in different modes (talk, writing, signs, visualizations, etc.).

### *Topic:* Experience-based knowledge

Knowledge, expertise, and communication are interacting components in a healthcare situation. Healthcare can be described as the purposeful use of biomedical knowledge, methods, tools, and skills to prevent, analyze, solve, or mitigate health problems. In most contexts, achieving favorable health outcomes hinges on effective communication between the professional and the patient. The Calgary-Cambridge model is a widely used model for teaching how to achieve purposeful communication in meetings with patients (Kurtz et al., 2016). It emphasizes on the purposeful use of nonverbal communication cues. It is not automatically given that the means of achieving effective communication in an analog health communication situation will work in a virtualized care environment. Likewise, a virtualized healthcare setting might allow for the use of other methods and tools to achieve the purpose of the consultation.

There are two principal means of developing healthcare knowledge. Experience-based knowledge arises gradually through clinicians’ thoughtful practice of healthcare and interaction with supervisors, peers, and supervisees (Fish & de Cossart, 2010). The experience-based knowledge that each clinician develops is subsequently applied in clinical practice. The other body of knowledge comes out of science. Both methods of developing knowledge require access to patients.

Knowledge is a commodity in knowledge-based care. Healthcare is a thoughtful analysis of health-related problems and the deliberate application of knowledge, skills, and tools to mitigate or solve the problem. In addition, healthcare helps patients to develop insights into the knowledge that explains their situation and the knowledge, skills, medicines, and tools that need to be applied to solve the problem.

### Topic: Knowledge-based practice

Healthcare professionals require know-how and know-what (Ryle, 2002; Sarangi & Gilstad, 2014). Know-how is the practical and procedural competence (De Cossart & Fish, 2005) necessary to conduct the task, such as management of a transducer in ultrasound examinations (Sarangi & Gilstad, 2014), or the handling of a syringe when injecting a vaccine. Know-what is propositional knowledge, including theories, concepts, and other kinds of knowledge referred to in journals and books. The community of practice develops know-how and know-what over time (Lave & Wenger, 1991). Relevant here is the operationalization of know-what in professional practice. The practice-field must critically embed new know-how and know-what in practice if this contributes to improving the service.

What knowledge to include in a research-based MOOC about eHealth is open to debate. In this project, a PhD-project contributed to inform the knowledge production within the team. Empirical data were gathered to investigate how healthcare professionals (in this case, nurses) and patients use and develop eHealth knowledge and eHealth literacy in their dialog. By looking at nurse-patient consultations in a highly digital clinical setting, examples from the data material can show us how patients and nurses share knowledge, and how nurses use their professional expertise to decide what information is relevant in the setting.

### Topic: IT health technologies

Although healthcare practice can be characterized as highly communicative (Toussaint & Coiera, 2005), health information technologies (HIT) are seldom designed to support communication or informed by theories of communication practice. The focus of HIT is on information processing, such as clinical decision support systems, and information sharing and exchange, such as electronic patient record systems. From the discussions in the interdisciplinary expert group, it became clear that the following questions must be taken into account while designing and implementing successful HIT for supporting communication:

1. How does HIT affect and is affected by communication practice?
2. What role does HIT play in the generation and sharing of clinical knowledge?
3. How does HIT integrate in clinical practice?
4. How can visual communication be used, as a complement to text, to support communication?

These four important questions were addressed in the dialogues between the experts involved in the development of the MOOC. These dialogues deepened the understanding of how social practice and technology co-design each other.

### Topic: Visual communication

Visual communication impacts patients and personnel in the healthcare context through a vast visual landscape that includes imagery, illustration, videography, graphics, symbols and icons, end-user apps, and digital tools with user interfaces (UI) and user experiences (UX). On top of this, the internet provides a constant stream of visual communication and design in health that present varying degrees of quality. Design is a tool, but what kind? A pioneer of graphic design, Paul Rand, defined 'design' as the relationship between form and content (Kroeger & Rand, 2008). While Rand's definition seems simple, the health context increases the need for solution-oriented visual tools. From a patient’s perspective, visual aids can be combined strategically to create optimal health messages, where infographics work best for short-term frames and text-based information works best for longer frames (Helsedirektoratet, 2021). According to Lyon (2016), drawing may help to ease communication regarding topics that are difficult to express verbally, while visual representations of health may benefit both patients and healthcare professionals. While designers have much to learn about interface design for health and healthcare, well-designed tools for patients and their caregivers are improving patients’ lives across all ages (Shneiderman et al., 2013).

Visual communication devices are useful, particularly early in a project, as they help to identify aspects of the project. Early in this collaboration, a semi-structured, actionable design process was initiated, which arose from the need to map the project visually. This mapping exercise was originally intended to give the designer a bird’s-eye view of the project’s scope and understand the various areas involved. The map was helpful in creating a visualization of various aspects, including each expertise area, the people involved, initial areas of focus, and early brainstorming ideas (Fig. 1).

## Results

### Results/ deliverables from the main project

The objective of the main project was threefold: 1) to develop knowledge and methods to assess the communicative practices of health personnel in relation to use of digital tools in the communication with the patients, 2) to develop knowledge and methods to assess patients’ eHealth literacy, and 3) to develop a knowledge based digital educational program in smart digital health communication (Table 4).

**Table 4:** Results/ deliverables from the main project

Results/deliverables	Sub-results	Reference
**Research**		
Theoretical development	Definition	What is health language? (Gilstad, 2021)
Analytical development	Discourse analysis of face to face- and digital communication in rheumatology settings	PhD (Larsen d 2022) Master thesis (Rian 2018)
Methodological development	Interdisciplinary dialogue	(This paper)
	Visualization tool (mandala)	(**Skogen**, 2021) Fig. 2, page 20
**Education and dissemination**		
Interdisciplinary MOOC about smart digital health communication	Five educational modules	https://digit.ntnu.no/courses/course-v1:NTNU+SDH_101+2022/course/
	Conference presentations and popular science	ALAPP, COMET, radio

### Interdisciplinary dialogue

The ongoing work with the deliverables in the main project informed the interdisciplinary dialogue. We consider the interdisciplinary dialogue itself represented a deliverable, although dialogue is not easily measurable. The output from the interdisciplinary dialogue was a valuable innovation, that went beyond the individual contributions of each individual and resulted in extended knowledge, not only for the individual but for the discourse communities concerned with digital health communication.

### A flexible design tool

The original map from the first visualization process (Fig. 2) quickly morphed into a new visualized representation: a platform for common reference and shared understanding between team members that became known as the “mandala” (Fig. 3). This flexible design tool allowed the team to progress into a set of sequential design steps to meet the deadline for the completion of the MOOC. The steps included reflective content-driven dialog, expanded learning and knowledge-sharing, adaptation to new directions, brainstorming, in-person workshops, idea iteration via synchronous group sessions, and asynchronous individual work followed by regular updates.

As the collaboration deepened, the mandala was used to continue to delineate areas of concentration to understand their relational context. It formed a consistent frame of reference through the different phases of the project. After numerous iterations, the relationships across the five areas of expertise were clarified, which resulted in the finely tuned descriptive “bridges” between each discipline. The color-coding solution was a carryover from the original map, and this continued to help in keeping the topics and subtopics distinct. Overall, the mandala is a simple visual representation. However, it packs a great deal of information in a condensed form.

**Fig. 4:**
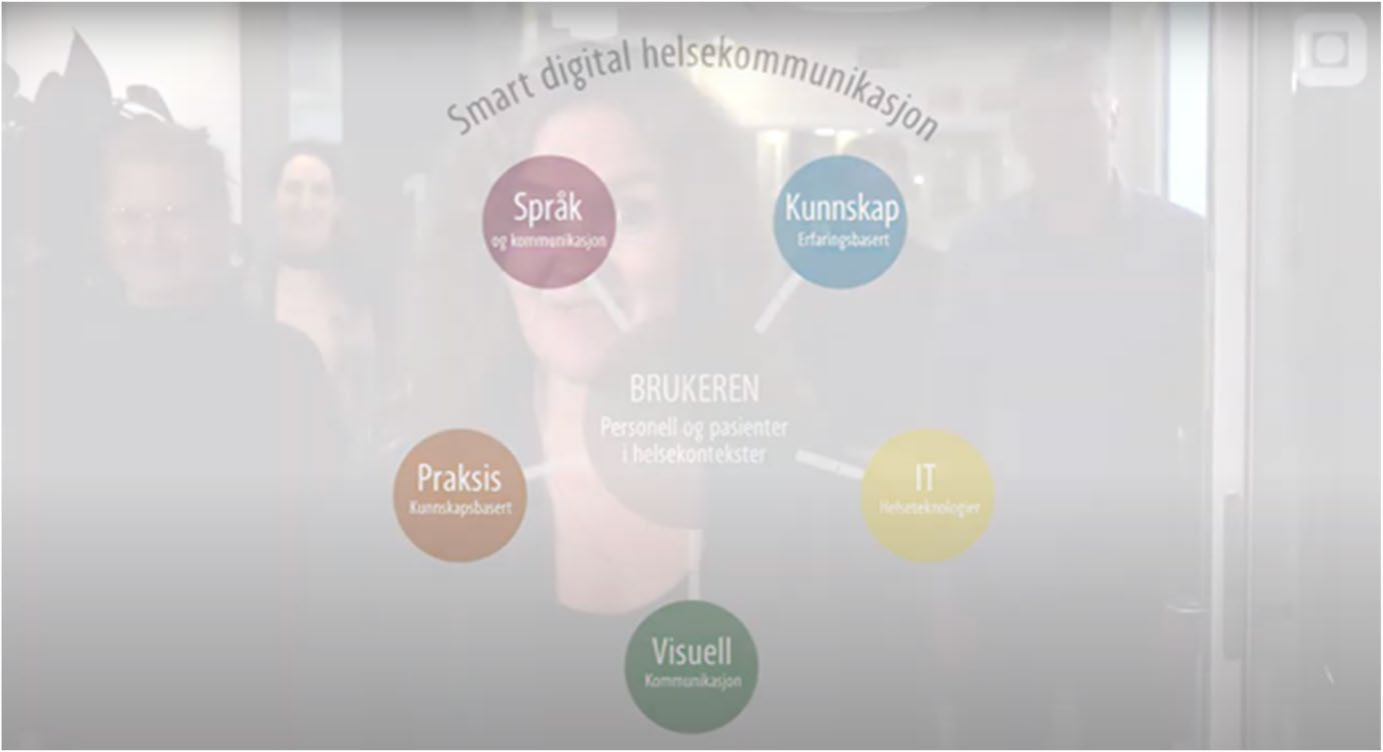
Example of frame from video in the MOOC showing how the mandala served as a mnemonic, navigational, and informational device that bound expertise areas together into a cohesive whole. The example shows an animated screen capture of the Norwegian MOOC as it fades from white screen with mandala to film. (Martha Skogen and Multimedia Center, NTNU, 2019. Permission granted by the authors)

The mandala provided a manageable reference, particularly when focusing on developing materials for the MOOC. As it had been successful for internal use, it was incorporated into the visual presentation of the MOOC as well. The pedagogical content of the MOOC was structured in a five-module system, where each module incorporated the mandala. In the MOOC context, the visual information became more than a framework for collaboration—it evolved into a meaningful, multi-layered device for course takers (Figs. 3, 4). As this MOOC could be experienced non-sequentially, the mandala served many functions, including being a:

- visual device for navigating the MOOC course material non-sequentially,
- mental map with mnemonic color codes that follow universal design principles,
- highly distilled, informational pedagogical tool,
- memorable, animated point of interest, and
- way to authenticate the course and make the information (and thus course itself) distinctly recognizable.

## Discussion

Schön (1983) suggested that reflecting on own professional practices resulted in learning and improved work. Reflecting- in- action is a creative process that includes improvisation and problem-solving as the work proceeds, while *reflecting-on action* is the ability to take a few steps back an assess reflectively the process and the work.

The conceptual considerations made during the project, as shown in the conceptual framework in Fig. 3 (‘mandala’), originated from scientific disciplines but were co-created and included an attempt to create conceptual and topical bridges between the disciplines. The knowledge and expertise applied included five scientific professionals with different epistemological backgrounds, knowledge objectives, theoretical and methodological approaches, and different levels of know-how and know-what. In the discussions, the participants drew on diverging scientific discourses, and applied scientific communicative competence to adjust to the discursive complexity. The discussion below is consequently twofold, including the conceptual framework and the expert communication in the interdisciplinary team.

### The conceptual framework

Visual artefacts were meant to develop a shared understanding in the multidisciplinary team. Common understanding was crucial to achieve a MOOC-design that reflected the perspectives of all team members. The conceptual framework included the respective scientific discourses and pedagogical approaches as well as the *mandala*. The conceptual framework provided insights into the value of interdisciplinary work over time, expanding professional vision by challenging established mindsets and practices to engage in a dialog with others. The mandala illustrated at least two levels of interdisciplinary insights: (1) it constituted a conceptual framework with topics for the MOOC regarding eHealth communication and (2) it was an illustration of the scientific conceptual framework that resulted from interdisciplinary expert dialog. An expansive conceptual framework may be a helpful organizing principle to other eHealth projects, as it shows the considerations of disciplines required for solving such complex tasks as developing eHealth solutions.

### The expert communication in the interdisciplinary team

As demonstrated in the results, the phenomenon of eHealth communication can be analyzed through different scientific lenses (language and communication, expertise, practice, design, IT), and activating different focal topics (see bridges in mandala, Fig. 2).

The scientific expertise required for the development of a MOOC about eHealth communication must involve scientific expertise of health (practices and systems), design (processes and interfaces), technology (system and functions), and language and communication (human interaction and contexts).

The success of collaboration depends on the mutual ability to adjust and internalize knowledge from the respective disciplines, while expressing own professional and scientific insights. This *scientific communicative competence* includes the ability to *contextualize* the activities within the institutional and professional frameworks, to *share* relevant professional and scientific insights that are relevant for the particular topic or subtopic, to *adopt* the insights of the other team members, to *internalize* others’ insights, and to *communicate* insights from one’s own and other scientific disciplines at an advanced level in interaction with others in the team.

### Literacy artefacts as team communication support

The literacy artefacts (Pahl & Rowsell, 2010) of the interdisciplinary team may support the interdisciplinary teamwork. In this study, the design of the process (Fig. 1) and the mandala (Fig. 2) became important literacy artefacts for the team, as they contributed to mutual understanding and constituted frames for the interdisciplinary reasoning and distribution of ideas and tasks. Choices about the content of the literacy artefacts were made in the group according to the needs and purposes. This demonstrates that literacy artefacts are not stable genres, but dynamically co-constructed.

Literacy artefacts are context-dependently multimodal. An example is the MOOC, which affords several modalities. As we saw in Fig. 3, the images and design constitute literacy artefacts for navigation and learning in the MOOC.

### Communicative strategies and practices

A team is composed of not only different scientific professionals but also different personalities. Personal qualities have an impact on collaboration and the atmosphere in the group (Wheelan, 2017; Johnson & Johnson, 2017). It is fundamental to establish a *safe space* for scientific communication. Creating a safe space for scientific communication and sharing of ideas in the team, while simultaneously adhering to contextual expectations, is important and should be a mutual responsibility of the team members. A communicative strategy may include conducting regular *meta discussions* regarding how the team is proceeding in terms of collaboration and communication and how each team member contributes to solving the tasks throughout the process, followed by establishment of norms and strategies for communication in the particular team. In such meta discussions, the following questions may feed the reflections: What is important when it comes to bringing different people, roles, etc. into the group? What personal qualities are important? How can we invite others into the group communicatively? How do we encourage creative, communicative space in the group?

### Significance in the eHealth context

Development of ehealth solutions require a complex set of skills, knowledge and experience in all phases, from idea, design, to development, delivery and use. It is not always easy to collaborate across the various expert and scientific disciplines. The reflections on how this interdisciplinary team exchanged knowledge and expertise across professional research fields, may serve as an inspiration to other teams aiming at developing eHealth solutions. Hopefully the mandala, the theoretical insights, and our collective experience offer important insights into the process of interdisciplinary teamwork. Succeeding in merging scientific knowledge may eventually benefit the end user, i.e., the health professional applying the MOOC, and subsequently their patients. Our common endeavor in developing the MOOC may be of significance for respectively healthcare professionals and patients. Healthcare professionals who want to deliver quality healthcare can benefit from the team’s common knowledge development. Patients can benefit form HCPs having greater understanding of their needs regarding ehealth systems.

The development of teaching and digital training solutions for eHealth requires interdisciplinary dialog that is founded upon openness and respect for other scientific insights and methodological approaches. This allows for time and room for the team members to integrate central scientific notions from relevant fields. As experts convene to collaborate in building solutions for the vast range of eHealth needs, interdisciplinary teamwork can also be rewarding as a shared learning experience.

The demands for high quality digital educational tools and learning activities are increasing in the education sector. In order to meet these demands, it is important that we gain experience from the development and use of existing educational tools. Development of educational tools requires both professional, pedagogical and practical-administrative knowledge and skills, as well as an adequate adaptation to the system and the users we want to address.

### Limitations of the study

A qualitative, retrospective analysis of the experiences of the team process may be illusory and simplistic. Past conflicts and power structures may be concealed. When writing the paper, the first author drove the development, and all participants in the team contributed to discussions and writing. Thus, potential misrepresentations have been addressed. However, there were some limitations. A limitation to the inter- and transdisciplinary approach is the reduced possibility of profoundly dealing with the topics’ respective scientific discourses over time. Professional education provides an opportunity to immerse into topics that are not possible in a project-based interdisciplinary setting. Thus, the task *and competence* of translating key points from one’s own discipline is crucial. Other limitations lie in the institutional and professional frameworks in which the project was developed, including funding structures that impose perspectives on behalf of others. Within these frameworks, researchers involved in smaller research areas may struggle to be heard. Likewise, a novice may experience more challenges in being heard in the team setting. The scientific disciplines represented by the team may present a limitation. Additional competencies, such as ethics and socioeconomics, would provide other insights. Finally, this work is informed by practices in the Norwegian eHealth system. The users of the MOOC are healthcare professionals, who are familiar with both social practices and ehealth solutions within this system. Other cultures or societies might need a different approach, and a different combination of professional, scientific and cultural background. These and other considerations should be addressed in the next phase of the project.

## Summary and conclusions

In this project, the interdisciplinary team developed a conceptual framework for an eHealth MOOC and used the visualized version as a communicative tool, including language and communication, practice-based knowledge, knowledge-based practice, design, and IT. The development of eHealth solutions requires interdisciplinary dialog and collaborative reasoning, including openness and respect for other scientific insights and methodological approaches, allowing the connection of central scientific notions from relevant fields while establishing a common understanding that leads to a meaningful whole. This is fruitful for assessing team communication. Visual communication may support dialog. Yet interdisciplinary teams may encounter disagreement or confusion owing to different scientific visions, epistemological points of departure, theoretical and methodological approaches, scientific genres, and terminologies. At such times, it is useful to clearly assess the status, achievements and goals of the interdisciplinary team with both reflections-in action, and reflections-on-action. Visual communication devices such as the mandala can help by providing a reference for common understanding and knowledge contextualization. It is hoped that this will serve as an inspiration for other interdisciplinary projects that aim to produce pedagogical material for higher education.

## Data Availability

Data is collected, managed, and analyzed in accordance with GDPR. The project is approved by the NSD - Norwegian Centre for Research Data.

## Declarations

This manuscript has not been published or presented elsewhere in part or in entirety and is not under consideration by another journal. We have read and understood your journal’s policies, and we believe that neither the manuscript nor the study violates any of these.
